# Sheehan syndrome: Cardiovascular and metabolic comorbidities

**DOI:** 10.3389/fendo.2023.1086731

**Published:** 2023-01-20

**Authors:** Bashir Ahmad Laway, Mohammad Salem Baba

**Affiliations:** Department of Endocrinology, Sher-I-Kashmir Institute of Medical Sciences, Srinagar, Kashmir, India

**Keywords:** Sheehan syndrome, cardiovascular disease, obesity, metabolic syndrome, insulin resistance

## Abstract

Sheehan syndrome (SS) caused by postpartum hemorrhage leads to partial or complete pituitary hormone deficiency. In addition to lipid and glucose abnormalities, patients with SS have increased body fat, insulin resistance (IR), coagulation abnormalities, increased leptin concentration, low-grade inflammation, and endothelial dysfunction that predispose them to cardiovascular diseases. Untreated growth hormone (GH) deficiency, hypogonadism, and excess glucocorticoid use are considered risk factors for these abnormalities. Compared to other hypopituitary subjects, patients with SS are younger and have a longer duration of disease and severe GH deficiency. Replacement with GH in addition to standard hormone replacement improves their cardiometabolic profile.

## Introduction

Sheehan syndrome (SS) also known as postpartum pituitary necrosis, though rare in developed countries, is one of the common causes of hypopituitarism in developing nations (1). It is primarily caused by vasospasm of hypothalamic portal vessels following massive postpartum hemorrhage (PPH), resulting in complete or partial loss of anterior pituitary gland cells. In addition, small sella turcica size, coagulation abnormalities and the presence of anti-pituitary antibodies contribute to ischemic damage to pituitary gland. Somatotroph and thyrotroph cell loss occurs in almost all patients, with preservation or recovery of gonadotroph, lactotroph, and corticotroph cell function in few (2–4). The duration from the onset of disease to diagnosis usually ranges from 7-19 years (2, 3). SS presents with long-standing non-specific symptoms of fatigue and generalized body aches. The major clinical features of typical and complete SS include lactation failure, failure of resumption of menstrual cycles after the puerperium, loss of pubic and axillary hair, symptoms of hypothyroidism, and hypocortisolism (1, 5). Less common manifestations of SS include hematological abnormalities (like anemia and pancytopenia), cardiac abnormalities (like cardiomyopathy and ventricular arrhythmias), and neuropsychiatric abnormalities (like psychosis) (6–9).

Metabolic abnormalities in patients with SS are a recent focus of attention. Patients with SS have increased body fat, insulin resistance (IR), dyslipidemia, coagulation abnormalities, increased leptin, low-grade inflammation, endothelial dysfunction (ED), and non-alcoholic fatty liver disease (NAFLD) (6, 10–14). Many of these effects are attributed to low insulin-like growth factor-1(IGF-1), hypogonadism, untreated secondary hypothyroidism, and glucocorticoid (GC) overuse (11). These conventional (age and dyslipidemia) and non-conventional (increased inflammatory markers and leptin) risk factors promote cardiovascular (CV) diseases in the general population as well as in hypopituitary patients like those with SS (15–19). Studies published in late 1900, which also recruited patients with SS, suggested that hypopituitary patients have increased mortality than the general population and predominantly died of CV diseases with an overall standardized mortality ratio (SMR) of 1.99 (15, 20, 21). Onset of hypopituitarism at a younger age and female gender were associated with higher SMR (20).

## Epidemiology of SS

The prevalence of SS is variable. In a population based study from Iceland, SS was diagnosed in 5.1 individuals per 100,000 population (22), whereas the prevalence of SS among 11,700 women >20 years of age was around 3% in northern India (23). In developed nations, up to 6% of hypopituitary subjects are diagnosed with SS (15, 24) while in countries like Turkey and Pakistan, up to one out of three cases of hypopituitarism is attributed to SS (25, 26). The lower prevalence of SS in developed countries is a result of better obstetric care facilities in these countries and possibly disease unawareness and missed diagnosis.

## Risk factors of CV diseases in hypopituitarism and SS

Hypopituitary patients, including persons with SS have higher mortality rates than the general population, which is attributed to increased CV disease, strokes and malignancy (15, 20, 27). The risk factors for increased mortality include younger age at diagnosis, female gender, diagnosis of craniopharyngioma, radiation therapy, transcranial surgery, diabetes insipidus, and hypogonadism. Dyslipidemia, ED and radiation induced vascular damage predispose these patients to higher risk of CV diseases (17, 28). Untreated GH deficiency (GHD) leading to IR, dyslipidemia and ED is the primary driver of increased CV mortality in hypopituitary subjects, who are adequately replaced with GC, thyroid and sex hormones (15, 28). In addition over-replacement with GC (29, 30) and sex steroid deficiency contribute to impaired metabolic parameters and atherogenesis in such patients (15). In a large study, atherosclerotic plaques in carotid arteries were present in half of the patients with hypopituitarism (31). Coronary flow reserve (CFR), as measured by transthoracic color echocardiography is a simple measurement of blood flow in coronary arteries, is impaired in subjects with GH deficiency, and corelates with serum IGF-1 concentration (32). Coronary artery calcification (CAC), a surrogate of coronary atherosclerosis was documented in around 50% of hypopituitary patients (33).

Compared to other hypopituitary subjects, patients with SS are younger in age, have longer duration of hypopituitarism, have severe GH/IGF-1 deficiency and decreased lean body mass (34). Though no long term data is available on CV mortality in SS, a large series of hypopituitary patients (which also enrolled few patients of SS) observed an increased mortality and morbidity primarily related to CV diseases in such patients (20, 35, 36). Two recent studies have demonstrated high frequency of coronary calcium deposits in women with SS. In one study enrolling 30 patients and an equal number of age and BMI matched controls, CAC score of >10 was documented in 32% of women compared with age/BMI matched controls (37), while in another study enrolling 60 patients of SS and 35, age and BMI matched controls, CAC score of >10 was documented in 27% of patients against 1.6% in controls (38).

## Obesity and dyslipidemia

As is true with hypopituitarism of other etiologies, patients with SS in comparison to age and gender matched population have increased body mass index (BMI) and total body fat with predominant abdominal fat deposition. Obesity and increased fat mass persisted after replacement with thyroxine and GC (10, 11). GH/IGF-1 deficiency appears to be the predominant contributor for abdominal obesity in such patients. GH impairs generation of active cortisol from inactive cortisone by inhibiting 11β-hydroxysteroid dehydrogenase type 1 (11β-HSD1). In presence of GHD, up regulation of 11β-HSD1 enzyme contributes to increased conversion of inactive cortisol to active cortisol. This phenomenon results in marked weight gain in hypopituitary patients with GHD even on small doses of GC (39). In one study, patients with SS had higher fat mass compared to other hypopituitary patients despite similar BMI, and waist circumference (WC) (34). Inspite of thyroxine and GC replacement, patients with SS have adverse lipid parameters like increased serum total cholesterol (TC), low density lipoprotein cholesterol (LDL-C) and triglycerides (TG) and low high density lipoprotein cholesterol (HDL-C). These lipid abnormalities are attributed to persistent GHD (10, 13) and replacement with GH improves these lipid abnormalities (11, 40).

## Metabolic syndrome and insulin resistance

In a large population of hypopituitary patients (not on GH replacement), half had BMI ≥30 kg/m^2^ and 86% had central obesity (defined as WC ≥94 cms in men and ≥90 cms in women). Around one-third were treated for hypertension and dyslipidemia (16). Similarly, a high prevalence of metabolic syndrome (MS) (50%) was observed in KIMS (Pfizer International Metabolic) database of 2479 patients with severe adult-onset GHD, naïve to GH replacement (41).

The prevalence of MS in SS is around 50% with major constituents being increased WC, decreased HDL-C and increased TG. Most of these abnormalities are a consequence of GHD, GC therapy and hypogonadism, all these factors favour abdominal fat deposition and atherogenic dyslipidemia. Around one fourth of patients with SS had diabetes mellitus and fasting and post-meal glucose values were high. Homeostasis model assessment-insulin resistance (HOMA-IR), an index of IR was high in large group of women with SS compared to healthy controls (10). Contrary to beneficial effects of GH replacement on lipids and body fat, glucose tolerance may worsen transiently after GH replacement and is attributed to insulin antagonistic effect of GH. The glucose increasing tendency of GH usually is greater in females and those receiving higher doses (11, 40, 42). The effect of GH replacement for 24 months on lipid profile, carotid intimal medial thickness (CIMT), glucose metabolism and visceral fat was studied in ten patients of SS and an equal number of controls matched for age and BMI. GH treatment had a favourable effect on CIMT, lipids and visceral fat. Despite change in body composition there was a tendency towards development of abnormal glucose tolerance (40). In a study recruiting 91 patients with SS, and were treated with GH for 24 months, blood glucose increased after 1 year but returned to normal levels at 2 years of treatment (40). These studies are limited by relatively short duration of follow-up and long-term consequences are not known. In addition to GH deficiency, excess GC replacement worsens metabolic profile of hypopituitary subjects which increases their risk for CV diseases (43).

## Chronic inflammation and endothelial dysfunction

Chronic low-grade inflammation, documented by increased high sensitive C-reactive protein (hsCRP), considered as coronary artery disease (CAD) risk enhancer is a better predictor of risk of CV events than LDL-C (44). In a cross-sectional study that enrolled 53 women with hypopituitarism and 111 healthy control women, interleukin-6 (IL-6) and CRP concentration were significantly higher in women with hypopituitarism than in healthy controls and were attributed to GH and estrogen deficiency (45). In another study enrolling 47 hypopituitary patients and 37 age, gender and BMI matched controls had higher hs-CRP levels despite lower levels of blood glucose and insulin resistance (46).

Likewise, in a study enrolling 30 patients with SS, on thyroxine and GC replacement but GH naïve, had higher hsCRP concentration compared to the healthy controls which correlated to insulin, HOMA-IR, HDL-C and IGF-1 (10). This inflammation in hypopituitary states like SS is attributed to GHD, hypogonadism, obesity and excess GC use (45). It is hypothesised that GH directly stimulates anti-inflammatory cells by cytokine receptors and indirectly through central redistribution of fat as visceral adipocytes which in turn release pro inflammatory markers like interleukin-6 in circulation (47). Replacement of GH in women with SS decreases hsCRP independent of improvement in serum lipids and lean body mass (48).

Endothelial dysfunction defined as change in vascular endothelium from antithrombotic to pro thrombotic state is considered an early marker for atherosclerosis which is detected before structural changes in the vessel wall are apparent on angiography or ultrasound (49). ED can be quantified by measuring flow mediated dilation (FMD) of brachial artery, CIMT or by quantifying serum adhesion molecules like Intercellular adhesion molecule-1 (ICAM-1), vascular cell adhesion molecule-1 (VCAM-1) and fibrinogen (50). Smith et al. studied 32 patients with hypopituitarism and observed that FMD of brachial artery was decreased compared to healthy subjects and GH replacement for six months improved the FMD along with other parameters of arterial stiffness (51). Also serum ICAM-1 and E-selectin were higher in GH deficient hypopituitary subjects (n=52) than healthy controls (n=54) which negatively correlated with IGF-1 concentrations (52). In another study enrolling GH naïve 30 patients with SS and equal number of matched healthy controls, serum ICAM-1 and VCAM-1 were increased in SS patients compared to age and BMI matched controls (10).

## Non-alcoholic fatty liver disease

Overall recent trends suggest that hypopituitary patients have higher prevalence of NAFLD owing to GHD, low thyroid hormones and low sex steroids (53). The prevalence of NAFLD varies from 54-70% on ultrasonography (54, 55). Similarly, patients with SS have higher prevalence of NAFLD compared with age and BMI matched controls. In a recent case control study, Das et al. studied 60 patients with SS for steatosis and liver stiffness, as measured by transient elastography. Hepatic steatosis of different grades was seen in 63% (with 50% having severe steatosis compared to 30% of controls). The mean-controlled attenuation parameter (CAP) score, a measure of hepatic steatosis, was significantly higher in patients with SS compared with age and BMI-matched controls. BMI and GH deficiency were two strong predictors of steatosis. In two such patients with biopsy-proven severe hepatic steatosis, the GH replacement resulted in complete resolution of steatosis (14).

## Effect of treatment on CV risk factors

Like other patients with hypopituitarism, treatment of SS patients with GH results in significant improvement in lipid parameters, lean body mass, endothelial function, arterial stiffness and exercise capacity (18, 50, 56–58) resulting in improved mortality benefits (20). In one study, 14 patients of SS treated with GH for 18 months, resulted in improvement in body composition (like decrease WC and waist to hip ratio) and lipid abnormalities (like decrease in TC, LDL-C, TG and increase in HDL-C) (11). Similarly in another study enrolling 10 patients with SS, replacement with GH for 24 months lead to reduction in the relation ApoB/ApoA ratio, increase in HDL-C, decrease in CIMT and visceral fat (40). In another large study enrolling 91 patients with SS, GH replacement for 24 months (after adequate thyroxine/GC replacement), improved lean body mass, TC and LDL-C but no improvement in WC and waist hip ratio was detected (34). Duration of treatment for less than 12 months in patients with hypopituitarism suggest that GH replacement has significant benefits on lean body mass and lipid parameters, at the cost of modestly decreased insulin sensitivity, predominantly in men (42). However, studies with longer duration of GH replacement suggest that insulin sensitivity does not decrease after GH replacement (34, 59) and the acute worsening of IR may be related to higher dose of GH prescribed (60). Likewise, it was observed that GH replacement for 24 months improved liver enzymes and markers of fibrosis in patients with hypopituitarism and NAFLD (61). It has also been observed that sympathetic tone is decreased in patients with SS and GH replacement in these patients improves sympathetic tone and normalizes sympathovagal balance after 6 and 12 months of treatment (62). **Table 1** summarises the studies on metabolic abnormalities in SS.

**Table 1:** Summary of metabolic abnormalities in patients with SS.

Author	Study subjects	Parameter	Results
**Bhat et al. (** 10 **)**	30 patients with SS and 30 controls	Assessed BMI, lipids, glucose, ICAM-1, VCAM-1 and hsCRP	Patients with SS have higher prevalence of metabolic syndrome and glucose intolerance, increased TC, TG, LDL-C hsCRP
**Mir et al. (** 13 **)**	30 patients with SS and 30 controls	Compared lipids and serum leptin	SS patients had higher TC, TG. LDL-C, Leptin and lower HDL-C
**Singh et al. (** 37 **)**	30 patients with SS and 19 controls	Coronary artery calcification score	Increased coronary artery calcification in patients with SS
**Soares et al. (** 40 **)**	10 patients with SS and 10 controls	Assessed lipids, blood glucose, insulin resistance, CIMT	Increased TG, decreased HDL-C, increased insulin levels in patients with SS than controls
**Tanriverdi et al. (** 11 **)**	10 patients with SS	Effect of GH on BMI, lipids, CIMT	After 18 months, GH decreased BMI, WC, TC, LDL-C and increased HDL-C
**Das et al. (** 14 **)**	60 patients with SS	Controlled attenuation parameter (for steatosis) and liver stiffness measurement (for fibrosis)	NAFLD was present in 63% of patients, with 51% having severe steatosis

## Conclusion

In addition to presence of conventional risk factors for CV diseases, SS patients have increased body fat, higher inflammatory and ED markers, which are directly correlated to GH/IGF-1 deficiency. Replacement of GH may ameliorate some of these risks, though at the cost of increased glucose tolerance. However, GH replacement may not be easily affordable in developing nations. But efforts should be made by government with the help of other non-governmental organizations, to provide GH at reasonable cost.

## Author contributions

BL and MB designed the study and contributed to the manuscript. BL and MB wrote the manuscript and reviewed the pertinent literature. Both authors contributed to the article and approved the submitted version.
